# A Cellular GWAS Approach to Define Human Variation in Cellular Pathways Important to Inflammation

**DOI:** 10.3390/pathogens5020039

**Published:** 2016-04-26

**Authors:** Samuel I. Miller, Anu Chaudhary

**Affiliations:** 1 Department of Microbiology, Department of Immunology, Department of Medicine, Department of Genome Sciences, University of Washington, Seattle, WA 98195, USA; 2 Department of Microbiology, University of Washington, Seattle, WA 98195, USA; anuc@uw.edu

**Keywords:** GWAS, Hi-HOST, host, lymphoblastoid, pathogen, pyroptosis, SIRS

## Abstract

An understanding of common human diversity in innate immune pathways should be beneficial in understanding autoimmune diseases, susceptibility to infection, and choices of anti-inflammatory treatment. Such understanding could also result in definition of currently unknown components of human inflammation pathways. A cellular genome-wide association studies (GWAS) platform, termed Hi-HOST (*Hi*gh-throughput *h*uman *in vitro s*usceptibility *t*esting), was developed to assay *in vitro* cellular phenotypes of infection in genotyped lymphoblastoid cells from genetically diverse human populations. Hi-HOST allows for measurement of multiple host and pathogen parameters of infection/inflammation including: bacterial invasion and intracellular replication, host cell death, and cytokine production. Hi-HOST has been used to successfully define a significant portion of the heritable human diversity in inflammatory cell death in response to *Salmonella typhimurium*. It also led to the discovery of genetic variants important to protection against systemic inflammatory response syndrome (SIRS) and protection against death and bacteremia in individuals with SIRS. Our laboratory is currently using this platform to define human diversity in autophagy and the NLPR3 inflammasome pathways, and to define new components that can impact the expression of phenotypes related to these pathways.

## 1. Introduction

Human susceptibility to disease depends on complex interactions between genes and the environment [1,2]. While clinical genome-wide association studies (GWAS) have identified numerous gene variants that contribute to the risk of disease, the functions of many genes whose variation is statistically associated with disease remains hard to define [3]. Characterization of the cellular pathways influenced by common human gene variation can provide an important framework to understanding associations between genetic variation and disease. GWAS approaches that identify effects of human genetic variation on specific cellular pathways can also provide important mechanistic insight on effects of human variation in diseases, and we have previously employed this approach to identify common human variation that influences human outcomes in relation to bacteremia [4,5].

## 2. Cellular GWAS Approach to Understand Pathways in Human Disease

Dissection of the genetic factors that contribute to clinical outcome is a difficult problem, particularly for infectious diseases. This is because case-controlled studies of infectious diseases can be limited by the small number of infected individuals and complicated by variability in infectious dose, treatment, nutrition, and other factors. This problem can in part be bypassed by studying *in vitro* cellular phenotypes of infection in genotyped immune cells (Box 1), and gave impetus for the development of a cellular GWAS based approach in our laboratory that screens cells from various populations collected as part of the HapMap project [6].

Box 1 **Advantages of a cellular GWAS approach to understand pathways in human disease.**

**Cellular GWAS: A complementary approach to GWAS of disease**

- Reduces environmental variation due to differences in dose, infectious strain, medical treatment and other concomitant factors.
- Provides clues to mechanism.
- Allows for experimental follow-up in the same cells used for the screen.
- Allows for controlled, reproducible measurements of phenotype in the laboratory.
- Not restricted to diseases that have large, currently accessible patient populations.
- Accessible to small labs.
- Study cellular phenotypes of relevance to multiple diseases.

### 2.1. Hapmap Cells Provide a Relevant Model to Study Immune Responses

Genotyped HapMap lymphoblastoid cells (LCLs) have also been used by others to examine human variation in gene expression [7], chemotherapeutic sensitivity [8], and HIV invasion [9], among other phenotypes. LCLs are EBV-transformed B cells, a cell type of importance to immune responses, and specific B cell subtypes are implicated in protection against microbial sepsis [10,11]. Cell surface phenotypes and cytokine production in these immune cells are stable over numerous passages in culture, making them a useful laboratory tool for identification, validation, and characterization of effects of human variation [12]. Furthermore LCLs from populations of African, Asian, and European ancestry allow for the study of genetic contributions to diseases that are variably prevalent in different groups. While offering several advantages for a cellular GWAS platform, the current limitations of Hapmap cells lie in the total numbers of available cells and their transformation state with EBV; reported variation in EBV copy numbers need to be controlled for as covariates during analysis. In addition, it limits phenotypes to those which can be assayed in B cell derived cells. In the foreseeable future, this technology may be extended to other genotyped human derived cells that can be stably maintained in culture. Despite these limitations, using these cells, we have developed a flow-cytometric method, *hi*gh-throughput *h*uman *in vitro s*usceptibility *t*esting (Hi-HOST), which allows for measurement of multiple host and pathogen parameters of infection. This platform overcomes limitations associated with standard cell-based infection assays that typically involve microbe enumeration through lysis of host cells and counting of bacterial colony-forming units or viral particles; and are therefore not amenable to large-scale phenotyping and are more prone to error.

### 2.2. Immune Phenotypes Studied in Hi-HOST

Starvation and exposure to pathogens are significant and perhaps the greatest selection pressures on the human genome [13,14]. One consequence of this selection is the notably diverse infectious disease outcome in humans [15,16,17]. Using stimuli that mimic these very pressures, we have examined human variation in important immune pathways using the Hi-HOST platform. We initially employed this platform to study host interactions of bacteria, specifically, *S. typhimurium*, *S. typhi*, and *Yersinia entercolitica*. Using fluorescently-tagged bacteria to facilitate flow cytometric tracking, we have characterized human variation in macro-pinocytosis used by *Salmonellae* for invasion and studied the intracellular survival and replication of these bacteria. In addition, we have studied variation of cytokine interleukin 10 (IL10) release in response to infection by *Listeria monocytogenes*, *S. typhimurium* and *Staphylococcus aureus*. Polymorphisms in IL10 are associated with sepsis mortality [18], and family studies of first-degree relatives and analysis of twins indicate that heritable genetic factors underlie inter-individual differences in quantitative IL10 production [19]. Here we describe two additional pathways that have been screened using Hi-HOST; inflammasome activation and autophagy that are activated in cells in response to pathogens and starvation.

#### 2.2.1. The Inflammasome

The inflammasome is an intracellular multimeric protein complex that responds to pathogens and tissue damage by promoting cell death and pro-inflammatory signaling, by activation of pro-inflammatory caspases 1, 4, 5, and 11 [20]. Growing evidence indicates that the inflammasome plays an important role in the pathogenesis of acute diseases like pneumonia [21] and sepsis [22]. Inflammasome-regulated cytokines are important in the development of acute respiratory distress syndrome triggered by pneumonia, sepsis and other insults [23]. Mice null in key inflammasome components succumb to *Salmonella typhimurium* earlier and have higher bacterial loads illustrating the importance of this pathway in host-*Salmonella* interactions [24,25]. We have used the Hi-HOST methodology to explore inflammatory cell death, pyroptosis, in response to *S. typhimurium* and discuss some of our key findings from this screen of Hapmap cells in Section 3, Section 4 and Section 5. Detection of various *Salmonella* components in the host can activate more than one inflammasome platform, *i.e.*, NLRC4 (NOD-, LRR- and CARD-containing 4) and NLRP3 (NOD-, LRR- and pyrin domain-containing 3) [26]. The role of NLRC4 in pathology of human diseases has remained unclear until very recently, when gain-of-function mutations in the NLRC4 gene that cosegregate with distinct autoinflammatory syndromes in affected families have been reported [27,28]. Variants are marked by recurrent fever, notable gastrointestinal pathology, systemic elevation of the levels of inflammatory markers and spontaneous ASC (apoptosis-associated speck-like protein containing a CARD) aggregation in macrophages. The NLRC4 inflammasome has been shown to provide a novel form of cell intrinsic defense against *Salmonella* infection, involving extrusion of infected enterocytes from the intestinal epithelium [29]. Intrinsic differences in the NLRP3 inflammasome is also implicated in various autoinflammatory diseases [30], and therefore we have screened for human genetic variants that influence selective activation of this inflammasome by pore-forming toxin nigericin.

#### 2.2.2. Autophagy

**Figure 1:**
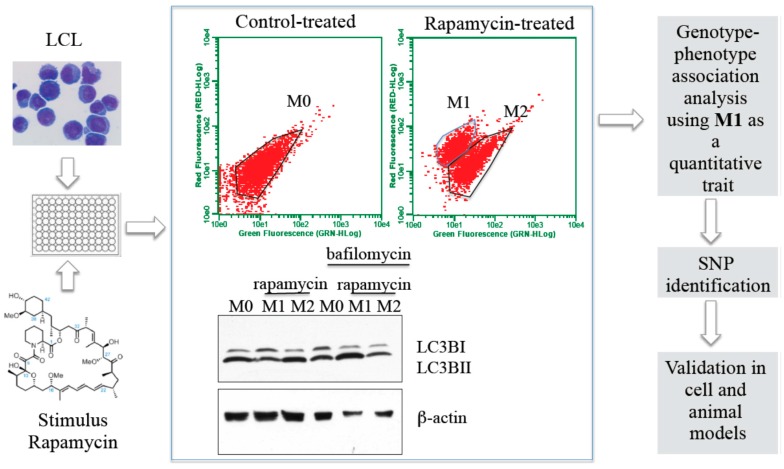
Schematic for the Hi-HOST autophagy screen. Genotyped lymphoblastoid cells (LCL) were treated with control buffer or rapamycin and then subjected to flow cytometric analysis after staining with LysoTracker. The two populations M1 and M2 in rapamycin treated LCL have differential autophagy as determined by western blotting. 500 LCL were thus screened and variation in the number of cells induced in M1 was used to perform genotype-phenotype association analysis using PLINK.

We have also extended Hi-HOST to measure the induction of autophagy in Hapmap cells in response to rapamycin (sirolimus), an FDA approved pharmacological inducer of autophagy. Autophagy is an essential homeostatic pathway in the host, and is important for clearance of cytosolic debris and bacteria. In addition, autophagy is important in thymic selection of T cells, survival of B cells, immune tolerance, and antigen presentation in the host. Figure 1 shows a schematic for the Hi-HOST autophagy screen for gene discovery that is being used in our laboratory. The ability of autophagy to clear bacterial pathogens was first demonstrated for *M. tuberculosis* [31] and group A *Streptococcus* [32]; and the interplay of bacteria with autophagy pathways has subsequently been shown for various other bacterial pathogens including *Shigella flexneri* [33] and *Salmonella enterica* [34]. Bacterial clearance through autophagy may be important in human disease, and marked autophagosome accumulation is noted in the livers of patients who die from sepsis [35]. Genetic deletion of critical autophagic proteins increases inflammatory responses in mice models of polymicrobial sepsis [36]. Autophagy is implicated in reduced ability to clear persistent UTI with uropathogenic *Escherichia coli* [37]. A role for autophagy in autoimmunity was first established with the identification of a mutation in an autophagy gene *Atg16L1* [38] in a GWAS for inflammatory bowel disease, and dysregulated autophagy is now implicated in various autoimmune disorders [39]. Autophagy also balances inflammasome activation to temper inflammation, suggesting a balance between these pathways may be critical during infection [40]. Human variation that trigger hyper- inflammasome or hypo-autophagy responses in humans are likely to provide useful insights into how inflammatory diseases and their outcomes vary within individuals [26].

### 2.3. Identification of Inherited Genetic Factors That Contribute to Phenotypic Variation in Inflammatory Pathways Using Hi-HOST

One key advantage offered by the Hi-HOST platform is the ability to understand the inherited contributor to phenotypic variation, since measures of phenotypic variance are dependent on combinations of biological and extraneous factors [41]. Significantly, a subset of the Hapmap LCLs are from parent-offspring *trios*, allowing us to estimate narrow-sense heritability, which captures only that proportion of genetic variation that is due to additive genetic values, by parent-offspring regression. As one might expect, there is considerable variation in the genetic contribution to phenotypic variance noted with the various immune phenotypes that we have examined in Hi-HOST. We determined that “heritability” of *Salmonella*-induced pyroptosis was 14.9% [4]; while for the phenotypes of *viable* invasion of *S. typhimurium* into cells and induction of rapamycin-mediated autophagy were significantly higher at 54% and 43% respectively (unpublished data). Significantly, with the identification of four genes, three of which are described below in Section 3, Section 4 and Section 5, Hi-HOST offers an explanation for around 7% of the variation in *Salmonella*-induced pyroptosis responses, thereby identifying around 50% of the genetic components of this variation, indicating its remarkable power. This analysis uses two combined population datasets, from the Yoruba in Ibadan, Nigeria (YRI) and Utah Residents with Northern and Western European Ancestry (CEU). It indicates that there is a genetic component that contributes to variability of these phenotypes observed in the laboratory that can be extracted and studied. The genetic differences identified in these screens have allowed for characterization of new biology of relevance to human disease. Here we briefly summarize some of our findings using this platform.

## 3. Expression of Non-Functional CARD8 Allows for More Robust Pyroptosis in Response to *S. typhimurium* Infection [4]

Early Hi-HOST screens demonstrated an association between more pyroptosis upon *S. typhimurium* infection and a SNP that resulted in a non-functional CARD8 [4], a negative regulator of inflammation. A comparison of CARD8 genes among different mammalian populations suggested that the increase in infectious disease burden associated with animals that live in herds or colonies may have naturally selected for loss of CARD8 multiple times in mammalian evolution. Loss of function of CARD8 is also more common among human populations that adopted agriculture earlier, and less so in populations that have traditionally lived as hunter-gatherers. These results suggested that loss of CARD8 may be one way in which a population evolves a more robust host response to deal with infectious diseases. On the flip side, a better ability to ward off infections may be associated with an increased risk for developing inflammatory diseases, and a link between CARD8 and severity of rheumatoid arthritis has been reported in multiple clinical studies [42]. We also found that loss of CARD8 was associated with a modestly increased risk in humans of the systemic inflammatory response syndrome (SIRS), suggesting an important link between the presence of CARD8 and the occurrence of sepsis.

## 4. A Role for Methionine Salvage Enzyme Apaf-1 (Apoptotic Protease Activating Factor 1) Interacting Protein (APIP) in Pyroptosis [5]

Hi-HOST has also identified a common genetic variant that is an Expression Quantitative Trait Loci (*e*QTL) for APIP in human cells and also contributes to human variation in *S. typhimurium*-mediated pyroptosis. *e*QTLs are genetic differences that correlate with expression levels of specific genes [7,43]. Hi-HOST led to the discovery that in addition to its role in apoptotic cell death by binding to apoptotic caspase, APIP is a component of the methionine salvage pathway and its reduction leads to the accumulation of 5′-methylthioadenosine. This in turn increases NLRC4-mediated caspase-1 activation. By linking methionine salvage pathways to pyroptosis in response to *Salmonella*, we identified important and unknown new biology and defined an important link between metabolism and immunity. The cell’s potential to balance cellular damage and bacterial clearance are dependent on the nutritional state of the host [3]. Significantly expression variation in APIP correlates with the odds of mortality in SIRS that are cut in one-half, indicating that the presence of different amounts of APIP may impact the outcome of bacteremic diseases.

## 5. A Role for Microtubule Stability in Pathogen-Mediated Pyroptosis [44]

A genetic variant that is an *e*QTL for tubulin β class 6, class V (TUBB6) also emerged in the screen for *S. typhimurium*-mediated pyroptosis. Overexpression of TUBB6 results in dramatic dismantling of the microtubule network in cells. We found that lower TUBB6 expression in cells correlated with more microtubule stability that increased *Salmonella*-induced pyroptosis. Besides providing interesting insights into the regulation of pyrotosis by microtubule stability, this study suggests that human variation affecting microtubule stability could also affect the drug responsiveness of microtubule-stabilizing and de-stabilizing drugs.

## 6. Summary

Starvation and pathogens together may be the biggest selection pressures on the human genome [13,14]. Given these drivers to human evolution, it is reasonable to evaluate their impact on our consequent immune responses in these very contexts. The Hi-HOST platform befits this query and offers unique insights into how common human variation influences responses to infectious agents and disease. It is a discovery tool for identification of new genes associated with specific pathways and can be used to investigate diverse phenotypes of relevance to human disease.
